# Unusual MRI findings of dural arteriovenous fistula: Isolated perfusion lesions mimicking TIA

**DOI:** 10.1186/1471-2377-12-77

**Published:** 2012-08-20

**Authors:** Yong-Won Kim, Dong-Hun Kang, Yang-Ha Hwang, Sung-Pa Park

**Affiliations:** 1 Department of Neurology, Kyungpook National University Hospital, Daegu, Republic of Korea; 2 Department of Neuroradiology, Kyungpook National University Hospital, Daegu, Republic of Korea; 3 Cerebrovascular Center, Kyungpook National University Hospital, Daegu, Republic of Korea; 4 School of Medicine Kyungpook National University, Daegu, Republic of Korea

**Keywords:** Transient ischemic attack, Perfusion-weighted imaging, Dural arteriovenous fistula, Magnetic resonance imaging, Transient ischemic attack mimics, Intracerebral hemorrhage

## Abstract

**Background:**

The diagnosis of transient ischemic attack (TIA) based on clinical history and objective findings, even including multiparametric MRI, can be misleading. We report two patients who presented with TIA-like deficits with isolated perfusion lesions in corresponding areas but were finally diagnosed as transient neurological symptoms associated with dural arteriovenous fistula (dAVF).

**Case presentation:**

Two patients presented with transient focal neurological symptoms lasting less than one hour. An isolated perfusion deficit with no diffusion change in the clinically relevant area was shown on brain MRI, indicating transient ischemia as the most plausible cause of neurological symptoms. However, cerebral angiography let to diagnosis of dAVF in both cases. Intracerebral hemorrhage occurred after the initial diagnosis of TIA in one patient, and the small area of perfusion abnormality accompanied by the enlarged cortical vein in the other case helped to identify the dAVF through the further investigation. The pattern of perfusion-weighted imaging in both cases revealed increase of mean transit time and relative cerebral blood volume denoting the venous congestion in a clinically corresponding area.

**Conclusion:**

Reported cases are uncommon clinical presentation of a dAVF, which can be misdiagnosed as TIA on clinical grounds. In rare cases, the isolated perfusion deficits could be attributable to venous congestion, despite the similar pattern of clinical presentation, such as with TIA.

## Background

The rapid assessment and management of transient ischemic attack (TIA) has emerged as the new standard paradigm due to evidence that patients with TIA are at high risk of recurrent strokes. [1] However, the accurate diagnosis of TIA based on clinical history and limited objective findings remains challenging, and a favorable outcome cannot be ensured for misdiagnosed patients.

Multiparametric MRI, including diffusion-weighted imaging (DWI) and perfusion-weighted imaging (PWI), is helpful in demonstrating the presence of ischemic injury and assessing the status of cerebral hemodynamics in patients with transient neurological symptoms. The yield of isolated PWI lesions, suggesting lone hypoperfusion, is reported to be 16% in TIA patients [2]. However, isolated PWI abnormalities could mask other underlying vascular pathologies. Herein, we report two cases of TIA-like deficits with isolated PWI lesions on the corresponding hemisphere that were confirmed as transient neurological symptoms associated with dural arteriovenous fistula (dAVF).

## Case presentation

**Figure 1:**
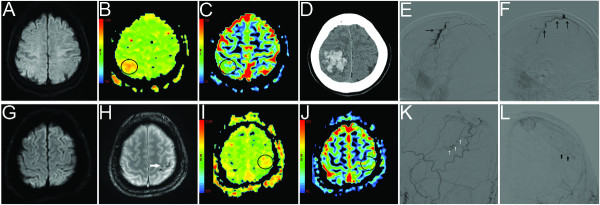
**Brain MRI, brain CT, and digital subtraction angiography (DSA) of the two cases.** Case 1. The baseline diffusion-weighted imaging (DWI) revealed no signal changes (**A**), while the perfusion-weighted imaging (PWI) showed a mean transit time (MTT) delay [interhemispheric ratio (IR) 1.07] and increase of relative cerebral blood volume (rCBV; IR 1.04) in the right postcentral gyrus (**B**, **C**). After neurological deterioration, a brain CT revealed a multiple lobar ICH in the right fronto-parietal lobe (**D**). A lateral view of the external carotid artery (ECA) on digital subtraction angiography (DSA) revealed a right middle meningeal artery-vein fistula with cortical venous reflux (**E**, **F**). Case 2. The baseline DWI showed no signal changes (**G**), whereas there were signal void signs in the left parietal cortex on the gradient echo MRI (**H**) and increase of MTT (IR 1.04) and rCBV (IR 1.24) on the PWI in the left paracentral sulcus area (**I**, **J**). Lateral (**I**) and frontal (**J**) views of the left ECA on DSA revealed arteriovenous fistula with single cortical venous reflux fed by the left middle meningeal artery.

Case 1) A 70-year-old woman presented to our emergency department (ED) complaining of one episode of left arm weakness accompanied by slurred speech and left hemifacial numbness approximately 40 minutes before the ED visit. These symptoms were spontaneously recovered 30 minutes after ictus. She had a medical history of hypertension and dyslipidemia and was taking only anti-hypertensive medication. At the initial examination, she was found to have no neurological deficits. At 2 hours from the onset of transient neurologic symptoms, a multiparametric stroke MRI (3.0-Tesla, Signa Excite, GE) showed increase of mean transit time (MTT) and relative cerebral blood volume (rCBV) in the right postcentral gyrus on PWI without any abnormal signals on diffusion-weighted imaging (DWI; Figures 1A to C). The gradient echo sequence (GRE) showed no signal voids, and the 3D time-of-flight MR angiography (3D TOF MRA) showed no intracranial vascular abnormalities. A risk factor workup, including transthoracic echocardiography, only revealed hypercholesterolemia (total cholesterol 226 mg/dl). During 5-day admission, she received antiplatelet and lipid-lowering agents without any recurrent events. One day after hospital discharge, she revisited ED presenting with newly developed left hemiparesis accompanied by slurred speech and vomiting. A brain CT showed a multiple lobar intracerebral hemorrhage (ICH) in the right fronto-parietal subcortex (Figure 1D), which necessitated an emergent digital subtraction angiography (DSA). A dAVF supplied by the right middle meningeal artery with the cortical venous reflux was found by a selective angiogram of the right external carotid artery (Figure 1E, F). An intra-arterial onyx embolization of the right middle meningeal artery was performed immediately. Two weeks later, a follow-up DSA revealed the disappearance of the direct arteriovenous connections, and the mild hemiparesis and hemihypesthesia of the left extremities remained.

Case 2) A 44-year-old man was admitted our department due to two episodes of right arm paresthesia approximately 14 hours and 3 hours prior to admission. These symptoms lasted for up to 5 minutes and spontaneously resolved without any treatment. On admission, neurological examination revealed no deficit. He had a medical history of hypertension, hypercholesterolemia and hypertriglyceridemia and was taking anti-hypertensive and lipid-lowering medication. A multiparametric MRI (3.0 T, Signa Excite, GE) performed 9.5 hours after the last episode showed perfusion abnormalities indicating the increase of MTT and rCBV (Figure 1I, J) in the left paracentral sulcus area without signal changes in the corresponding area on DWI (Figure 1G). The 3D TOF MRA also showed no intracranial vascular abnormalities. A signal void sign in the left parietal cortex on GRE, indicated vascular abnormalities (Figure 1H), and brain CT angiography was performed. An enlarged and tortuous cortical vein was found in the left parietal area. The dAVF with a single cortical venous reflux fed by the left middle meningeal artery was confirmed by DSA (Figure 1K, L). The next day, the patient underwent Gamma Knife radiosurgery. During the follow-up of 6 months, patients remained symptom free.

## Discussion

We reported two cases of dAVF that presented with transient neurological symptoms. For the clinical standpoint, these type of transient symptoms more frequently correspond to TIA or other conditions such as seizures, migraine, psychiatric conditions, toxic-metabolic derangements, or neuropathy [3,4]. In our cases, the clinical history of a brief episode of focal neurological dysfunction accompanied by the imaging evidence of a perfusion deficit in an appropriate area could have led to the diagnosis of transient neurological symptoms attributable to ischemic pathology. [5] ICH occurred after misdiagnosis in case 1, and the small area of perfusion abnormality accompanied by the enlarged cortical vein in case 2 helped to identify the dAVF through the further investigation. Unlike arterial ischemia, PWI revealed increase of MTT and rCBV denoting the venous congestion in a clinically corresponding area in both cases [6-8]. The DSA showed parietal dAVF supplied by the middle meningeal artery and draining to cortical vein in the area corresponding perfusion deficit on PWI.

The dAVF may present with a wide range of symptoms. Pulsatile tinnitus, audible bruit, and headache are usually benign. Neurological deficits including seizure, motor/sensory symptoms, cranial nerve palsy, visual disturbance, hearing loss, and others can be induced by regional decreases in cerebral blood flow, such as cortical venous reflux, venous congestion and retrograde venous drainage [9-11]. Intermittently increased pressure on engorged and congested veins can produce the transient neurological symptoms in dAVF [9-11]. The most serious presentation is the intracranial hemorrhage associated with retrograde cortical venous drainage [9,11-13].

The brain MRI in patients with dAVF reveals various findings depending on the pattern of venous drainage. In case of dAVF without retrograde venous drainage, MRI findings are almost normal. On the other hand, MRI of patients with dAVF with retrograde venous drainage or venous congestion can exhibit flow void sign on the brain surface. [12,13] Venous congestion may demonstrate medullary vein enlargement and high intensity of T2-weighted MRI in white matter [12,13]. Perfusion studies using MRI or CT are able to provide hemodynamic parameters. The CBV from among hemodynamic parameters correlate with retrograde venous drainage, which is useful method for assessment of dAVF with retrograde venous drainage [6-8]. The analysis of hemodynamic parameters may contribute to differentiate the pathologic mechanisms as follows; 1) decrease of cerebral blood flow (CBF), CBV and MTT in functional type of steal, 2) decrease of CBF, CBV with increase of MTT in ischemic type of steal, or 3) increase of CBV and MTT in venous congestion [6].

In rare cases, transient symptoms could be attributed to venous congestion, despite PWI findings similar to ischemic stroke. Our cases demonstrated that the isolated perfusion deficits in clinically relevant areas might be due to venous congestion from a high flow shunt, which corresponds with a transient clinical presentation.

## Conclusions

In cases presenting as transient neurological symptoms, a perfusion deficit in a clinically relevant area does not always indicate TIA or stroke. Although urgent diagnosis and management is important for patients with TIA, the attending physicians should consider other neurological conditions in assessing transient neurological symptoms.

## Consent

Written informed consents were obtained from the both patients for publication of this report and any accompanying images. A copy of the written consent is available for review by the Editor-in-Chief of this journal.

## Competing interests

The authors declare that they have no competing interests.

## Authors’ contribution

Study concept and design: YH Hwang. Analysis and interpretation of data: DH Kang, YW Kim. Drafting of the manuscript: YW Kim. Critical revision of the manuscript for important intellectual content: YH Hwang, SP Park. All authors read and approved the final manuscript.

## Pre-publication history

The pre-publication history for this paper can be accessed here:

http://www.biomedcentral.com/1471-2377/12/77/prepub
